# Fox-fordyce disease: Pulsed dye laser versus fractional Co_2_ laser treatment

**DOI:** 10.1016/j.jdcr.2023.08.025

**Published:** 2023-08-30

**Authors:** Abdulaziz Alnoshan, Mohammad Almazied, Lena Alghamdi

**Affiliations:** a Dermatology Division, Department of Internal Medicine, Security Forces Hospital, Riyadh, Saudi Arabia; b Dermatology Department, King Salman Hospital, Riyadh, Saudi Arabia

**Keywords:** Fox-Fordyce disease, fractional CO_2_ laser, pulsed dye laser

## Introduction

Fox-Fordyce disease (FFD) was initially documented by George Henry Fox and John Addison Fordyce in 1902.^1^ It is an uncommon form of skin inflammation affecting apocrine glands in the areas, such as axillae, genitalia, and pubic area. It manifests as numerous, itchy, skin-colored follicular papules. The exact etiology remains unknown; however, it has been linked with gender- and age-specific hormonal changes, with 90% of cases seen in women aged between 13 and 35 years.^2^ The results of histopathologic examination for FFD may vary and can include blockage of the infundibulum, excessive buildup of keratin on the skin surface, thickening of the skin, accumulation of fluid in the tissues, and presence of nonspecific inflammatory cells.^3^ Hence, diagnosis primarily relies on clinical assessment because histopathologic observations are frequently variable and lack specificity.^4^ At present, to our knowledge, no established treatment can definitively cure FFD. There are different treatment options, including topicals, oral medication, and lasers.^5^ Here, we present the case of a 28-year-old woman with FFD in both axillae that was managed with a pulsed dye laser (PDL) on the right axilla and a fractional CO_2_ laser on the left axilla.

## Case report

**Fig 1:**
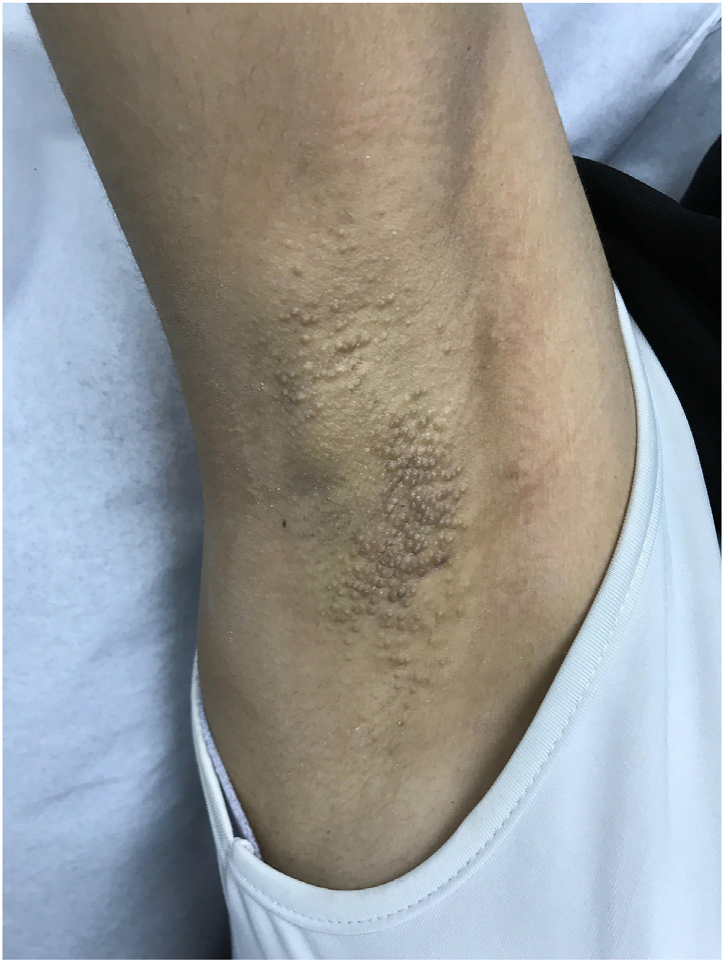
Right axilla before treatment showed multiple well-defined, round, follicular, skin-colored papules, 2 to 3 mm in diameter.

**Fig 2:**
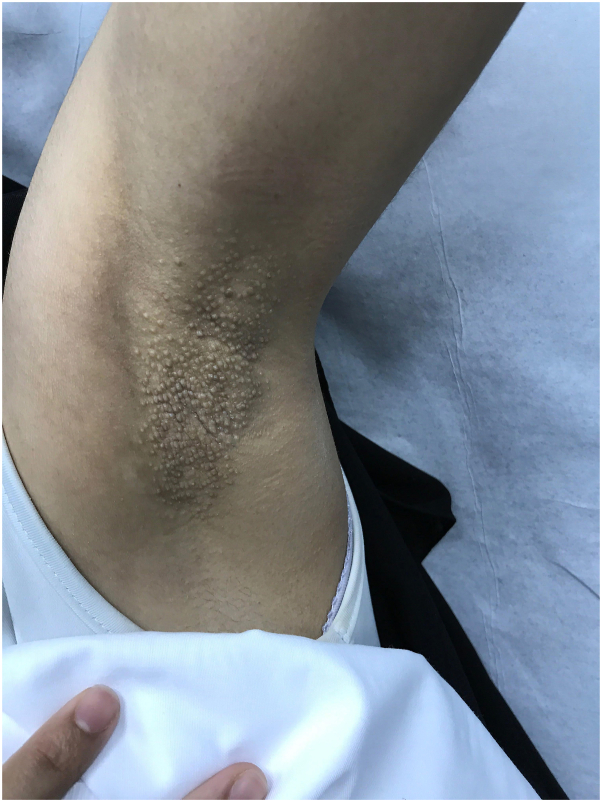
Left axilla before treatment showed multiple well-defined, round, follicular, skin-colored papules, 2 to 3 mm in diameter.

**Fig 3:**
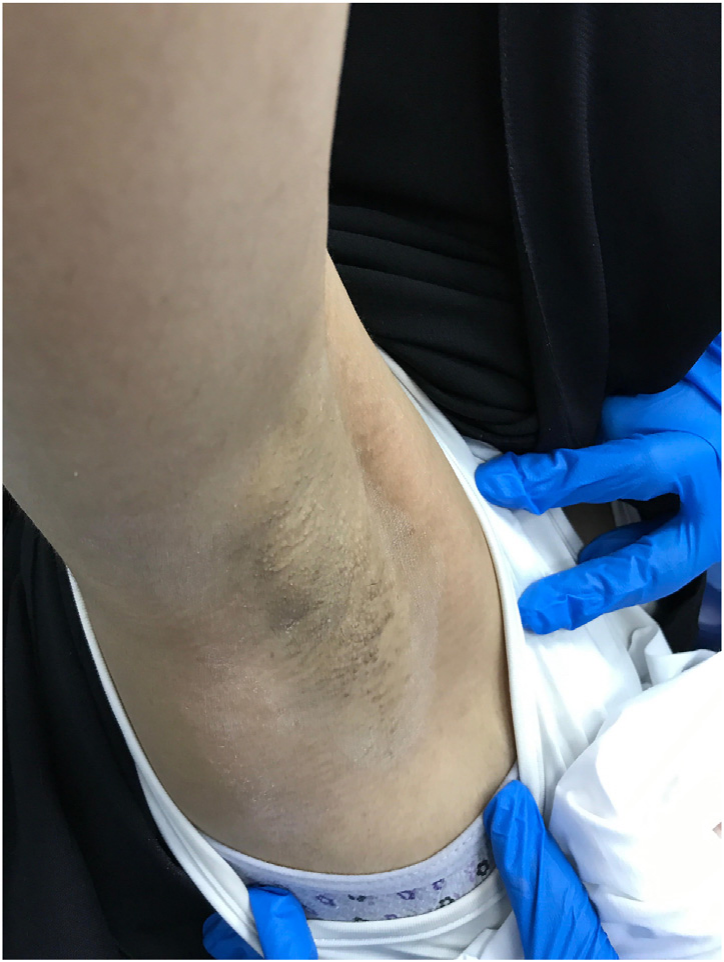
After the third laser session of pulsed dye laser, hair regrowth, and sweating were noticed in the right axilla.

**Fig 4:**
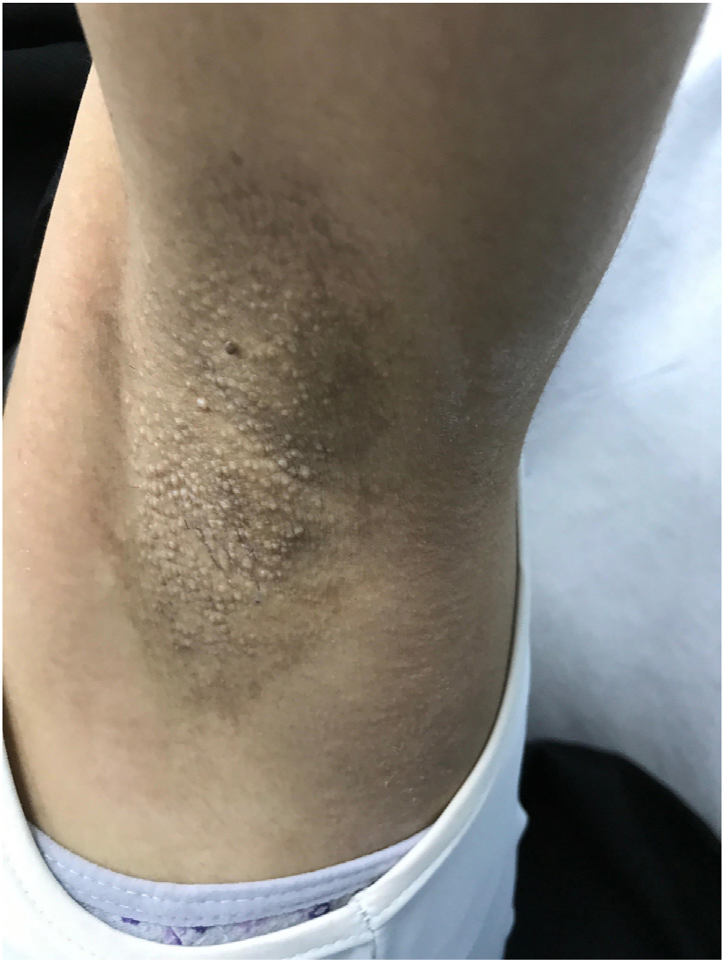
After the third laser session of fractional CO_2_ laser, hair regrowth, and sweating were noticed in the left axilla.

A 28-year-old woman presented to the clinic with multiple, itchy skin lesions over both axillae that have lasted for 4 years, increasing in number over time and becoming aggravated in summer. Previously, these lesions were treated with 1% topical clindamycin phosphate solution twice daily for 2 months with no improvement. Physical examination showed numerous well-defined round, follicular, skin-colored papules, 2 to 3 mm in diameter, over bilateral axillae (Figs 1 and 2). The hair in both axillae was spare with normal adjacent skin. A 4-mm punch biopsy was taken from the left armpit that revealed mild-infundibular follicular dilatation associated with keratin plugging. Furthermore, a mild-perivascular lymphocytic infiltrate with occasional eosinophils was observed. The treatment plan was 3 sessions of a PDL for the right axilla and a fractional CO_2_ laser for the left axilla with intervals of 6 weeks. However, the interval between the second and third sessions was delayed from 6 weeks to 5 months because of patient travel. The devices used were PDL Candela Vbeam Perfecta (model number 9914-08-0300) and Lutronic fractional CO_2_ laser (model eCO2 Plus). Regarding device settings, the PDL had a fluence of 8 J/cm^2^, spot size of 7 mm, 85 pulses, pulse duration of 3 ms, and level-2 dynamic cooling device; for the fractional CO_2_ laser, TIP 120 was employed, set at scan-type static, 60 mJ, 30 W power, and 100 spots/cm^2^ density. The end point for the fractional CO_2_ laser was white spots, while the PDL showed mild erythema because of nonpurpuric settings. After the third session, there was an improvement in the shrinking of papules in both axillae (Figs 3 and 4). However, the PDL showed better results than the fractional CO_2_ laser. The patient was followed up in the outpatient clinic for 6 months with no recurrence.

## Discussion

The management of FFD is challenging because there is no definitive treatment. Treatment varies from topicals, such as retinoic acid, clindamycin, pimecrolimus, and corticosteroid, to systemic treatments, such as estrogen-based oral contraceptives and isotretinoin, or even laser treatment by PDL or fractional CO_2_ laser.^5^ However, the success rate of topical and oral treatments is limited, with a high-recurrence rate.^6^ Regarding laser treatment for FFD until the February 11, 2023, only 3 cases were reported in the MIDLINE database; 1 reported using PDL, 1 reported using fractional CO_2_, and 1 reported fractionated erbium glass laser.

A systemic review regarding PDL treatment for inflammatory skin diseases showed good results in decreasing inflammation by altering inflammatory cell response. Because FFD is an inflammatory disease, we decided to use PDL.^7^ PDL treatment in FFD treatment was reported. Uzuncakmak et al^8^ (2016) reported a case of a 17-year-old female patient who presented with skin-colored papules with multiple follicles over both axillary fossae. The histopathologic examination revealed that the patient had mild eccrine sweat gland dilatation and fibrosis, perifollicular fibrosis, and chronic inflammation, which led to a diagnosis of FFD. The patient was initially treated with topical clobetasol propionate ointment with no benefits. After that, PDL application (585 nm) started with 8 J/cm^2^, and 6 weeks apart. Hair growth and sweating were noticed after the first session. After completing 7 sessions, the patient was almost completely recovered and was free of the disease for 1 year. However, recurrence of the disease started with milder severity compared with the first presentation.^8^

A fractional CO_2_ laser to treatment for FFD was reported in 2013, by Al-Qarqaz et al.^9^ It was a case of a 24-year-old female patient who had been experiencing itchy papules over both axillae for 7 years. Histopathologic examination revealed enlarged apocrine glands with mononuclear cell infiltration in the adjacent dermis. She received various topical treatments (including retinoids, steroids, and antibiotics), but none of them led to any improvement. After that, the fractional CO_2_ laser was started. Three sessions were completed, with power at 20 to 25 W, pitch at 600 to 400 μm, and dwell time at 500 to 900 μs with an interval of 6 to 8 weeks. There was a progressive improvement in skin lesions and itchiness. They had no relapse 3 months after the last session. However, although short-term outcomes have been reported, the long-term results could not be documented because the patient did not attend the follow-up appointments.^9^

Fractionated erbium glass laser’s use in treating FFD was reported in 2016 by Han et al^10^ who published the case of an 18-year-old female patient with multiple bilateral 3- to 4-mm, skin-colored, dome-shaped areolar papules on each areolae. The histopathologic report showed infundibular dilatation and hyperkeratosis. Initially, she was treated with surgical excision, followed by a 1550-nm fractionated erbium glass laser. Three laser sessions 1 month apart were conducted in 2 modes. Two passes were performed in stamping: mode at 50 mJ followed by one pass in moving mode at 25 mJ. The patient was free of the disease after a follow-up period of 14 months from the last laser session and was pleased with the outcomes.^10^

In conclusion, to our knowledge, this is the first case report comparing PDL and fractional CO_2_ laser for treating FFD in the same patient. The patient improved with both lasers; however, the PDL results were superior. Further studies on laser treatments for FFD need to be done.

## Conflicts of interest

None disclosed.
